# Digital Technology, Knowledge Level, and Food Safety Governance: Implications for National Healthcare System

**DOI:** 10.3389/fpubh.2021.753950

**Published:** 2021-11-25

**Authors:** Xun Xi, Shaobin Wei, Kuen-Lin Lin, Haitao Zhou, Kui Wang, Haiyan Zhou, Zhao Li, Nan Nan, Liping Qiu, Feng Hu, Fu-Sheng Tsai, Dongxiang Chen

**Affiliations:** ^1^Global Value Chain Research Center, Zhejiang Gongshang University, Hangzhou, China; ^2^School of Management, Shandong Technology and Business University, Yantai, China; ^3^International Business Research Institute, Zhejiang Gongshang University, Hangzhou, China; ^4^Department of Business Management, College of Management, Cheng Shiu University, Kaohsiung, Taiwan; ^5^School of Master of Business Administration, Zhejiang Gongshang University, Hangzhou, China; ^6^School of Economics, Huazhong University of Science and Technology, Wuhan, China; ^7^China Center for Economic Research, East China Normal University, Shanghai, China; ^8^School of Economics and Management, Shihezi University, Shihezi, China; ^9^Department of Business Administration, Cheng Shiu University, Kaohsiung, Taiwan; ^10^Center for Environmental Toxin and Emerging-Contaminant Research, Cheng Shiu University, Kaohsiung, Taiwan; ^11^Super Micro Mass Research and Technology Center, Cheng Shiu University, Kaohsiung, Taiwan; ^12^School of Business Administration, Zhejiang University of Finance & Economics Dongfang College, Haining, China

**Keywords:** digitalization, food safety supervision efficiency, knowledge level of producers, knowledge level of consumers, food safety regulation

## Abstract

Exploring the intrinsic relationship between digital technology and the efficiency of food safety supervision contributes to a better understanding of the role of digital technology in food safety supervision and how to maximize its influence. This study employed sample data from 31 regions in China between 2015 and 2017 for an empirical analysis of the correlation between the two and to examine the moderating effects of the knowledge levels of food producers and consumers. The results show that the development of digital technology contributes to enhancing the efficiency of food safety supervision. In this process, the higher the knowledge level of consumers, the greater the positive promotional effect of digital technology. On the contrary, when the knowledge level of producers is higher, it is not conducive to enhancing the effect of digital technology on the efficiency of food safety supervision. The author holds the view that this is related to the fact that employees in the food production and manufacturing industry have insufficient moral and legal knowledge. This not only limits the effect of digital technology on enhancing the efficiency of food safety supervision, but also opens the door to illegal production for some unprincipled producers. The policy implications are that digital technology should be used to improve food safety supervision, the moral and legal knowledge of food producers should be improved, and consumers should be encouraged to use digital technology more in the pursuit of food safety. Implications for national healthcare system would be also discussed in our paper.

## Introduction

As a fundamental factor in quality of life, food safety is crucial to people's lives and health. It is a matter of societal concern and something that governments find difficult to regulate. The lack of integrity of some food producers who are driven by self-interest and inadequate government supervision mean that the maintenance of food safety has become a global problem. According to the World Health Organization, approximately 600 million people suffer from foodborne diseases each year, of whom 420,000 die, resulting in a loss of 33 million healthy life years^1^ For example, 48 million people in the United States contract diseases from eating contaminated food every year, of which 128,000 are hospitalized and 3,000 die. The economic loss caused by foodborne diseases is approximately as high as 93.2 billion USD (1). Food safety issues have gravely affected human life and health and have caused great harm to society and the global economy.

To improve the quality of food safety supervision, an adequate accountability mechanism must first be built so that local governments, regulatory agencies, and manufacturers can have clear concepts of their roles within the accountability system, exert corresponding influence, and form a joint force. At the same time, it is necessary to improve food safety supervision with the intelligent use of technology and data. Few studies have explored the issues of digital technology and food safety supervision; therefore, we are unable to fully understand the role that digital technology plays in this process. This impedes the improvement of digital technology and its application to food safety supervision. To fill the gap left by existing research, this study intended to answer the following two questions: First, how does digital technology affect food safety supervision? Second, do the knowledge levels of producers, consumers, and direct stakeholders in food safety influence the effect of digital technology on the efficiency of food safety supervision?

It is certain that this study can help managers realize the key influencing factors to improve the efficiency of food safety supervision, so as to improve the efficiency of food safety supervision and maintain the health of consumers. Of course, this paper describes the impact of digital technology on improving the efficiency of food safety supervision, and emphasizes that digital technology plays an important role in reducing the occurrence of events damaging consumers' health.

## Theoretical Basis and Research Hypotheses

Food safety supervision usually refers to the oversight over food production and processing by the government and regulatory bodies (2). Efficiency can be considered as the ratio of output to input, including three categories: technical efficiency, allocation efficiency, and efficiency of scale (3). According to these definitions of food safety supervision and efficiency, for the purpose of this study, food safety supervision efficiency is defined as the ratio of the relationship between the cost invested by the government and food safety regulatory agencies in the process of implementing food safety regulatory actions to the regulatory output (i.e., regulatory results). Existing research on food safety supervision mostly applies the cost–benefit approach in the analyses. For example, Herman et al. (4) found that to decide on whether and how to implement food safety regulations, the cost–benefit approach is essential. Traill and Koenig (5) took the British government as the subject of research and introduced a cost-benefit approach that can be used to evaluate the efficiency of food safety supervision thoroughly. In addition, Millstone et al. (6) opined that the government should supervise food supply and improve the efficiency of food production supervision to ensure food safety. Han and Yan (7) believe that, considering the finiteness of regulatory resources and the problems of regulatory costs, a regulatory model that is led by the government and supported by enterprises should be constructed.

Digital technology generally consists of technologies such as blockchain, big data, cloud computing, and artificial intelligence. The biggest advantage of its application is that it can improve the overall economic efficiency of society. The problems of plane connection include excessive nodes and low efficiency. Digital technology can build a more direct and efficient network, breaking the plane connection between enterprises and enterprises, people and people, and between people and materials (8). Giacomo et al. (9) believe that a structure with amplified natures of multidimension and interaction will be established in the future through digital technology. The end-to-end interactive connection mode in this structure will eliminate intermediate nodes and further improve interactions and cooperation between subjects. In addition, the blockchain based on the digital technology will strengthen the trust of consumers and customers, maintain a state of low cost and high efficiency in economic operation, and drive the rapid development of society. The application of digital technology has formed a trend, especially for traditional industries such as food production. To break through the production frontier and improve product quality, the support of digital technology is indispensable.

### Digital Technology and Food Safety Supervision Efficiency

As the food supply chain continues to lengthen, regulators and consumers lack sufficient and accurate information to trace the authentic sources of food or locate the origins of food quality problems in time. Digital technology can bring about various approaches to improve the status of food safety supervision (10, 11), including the tracking of food safety information, improvement of relevant laws and regulations, and the enhancement of the spread of food safety knowledge.

The development of digital technology is conducive to the tracking of food safety information. In an ideal situation, the monitoring of food quality would involve the whole process without the loss of any information. Whole-process monitoring is difficult to achieve against the background of large-scale food production, distribution, and sales. However, with the development of digital technologies such as wireless sensing and the Internet of Things, food safety supervision can go beyond the constraints of the existing workforce and material resources and entail a system as close as possible to the real-time monitoring of food production and distribution. Cranfield et al. (12) applied radio frequency technology and blockchain technology to construct a conceptual framework of a traceable system of the food supply chain and analyzed the advantages and disadvantages of the application of digital technology and its approaches. Awuor et al. (13) further proposed a food safety emergency plan based on digital technology, linking all information in the food supply chain, and established an efficient and reliable execution environment so that when food safety issues occur, core problems can be traced rapidly and losses can be minimized. Shinwell and Defeyter (14) took meat as an application scenario for the construction of a model of a digitisation system and performed functional verification. Digital technology improves the efficiency of food safety supervision by government departments by realizing information tracing.

The development of digital technology is conducive to the improvement and application of relevant laws and regulations. Consumers can only obtain food safety information from publicly available data in the food market, which means that information access is asymmetric. Consumers will usually be unaware of safety risks if producers and processors deliberately conceal information about it. In addition, the shortfalls in the timeliness and accuracy of information disclosure of food safety supervision by government departments lead to a situation in which most consumers cannot obtain dynamic information of food inspection on time, which increases the safety risks. Because of identification difficulties and inaccurate judgments, loopholes in relevant laws and regulations may be exploited when these safety issues occur. Reeder et al. (15) believe that digital technology will significantly promote the informatization level of the food industry, increase the transparency of food safety information and the supervision efficiency of administrative agencies, and is a powerful tool for social co-governance. From a legal perspective, Marcotrigiano et al. (16) explored how to introduce digital technology into the supervision of food safety.

On the one hand, digital technology improves the efficiency of food supervision and reduces management costs. On the other hand, it reduces the rent-seeking behavior of government departments and protects consumer rights in some countries. By improving relevant laws and regulations, digital technology improves government departments' food safety supervision efficiency.

The development of digital technology is conducive to spreading knowledge about the safe production and consumption of food. The governments of various regions in China have formulated regulations on food quality standards, food contaminants, pesticide residue limits, and specifications of food hygiene practices. Although the overall level of the standard system is slightly lower than the average international level, the technical level and hardware conditions of Chinese food producers and processors do not meet the standard in many cases, and this is one of the factors that reduces the efficiency of food safety supervision. Digital technology closely connects all links in the food production and processing chain by building a network platform that combines the online and offline worlds and promotes the continuous circulation of advanced production and processing technologies within the food industry (17). It can increase the degree of codification of high-quality production and processing knowledge, making this easier to be disseminated and accepted (18). Digital technology can also strengthen the tacit understanding between the business partners of the food production and processing chain; therefore, any technical requirements of each link can be satisfied by matching suppliers in time (19). In addition, consumers can learn more about food safety through digital technology and promptly report food safety issues to producers and regulators during consumption (20). Digital technology improves the efficiency of food safety supervision of government departments through the spillover of knowledge in food safety production and consumption.

H1: There is a positive correlation between digitalization and the efficiency of food safety supervision.

### Producer Knowledge Level

In some cases, high-level technology and equipment are needed for the safe production of food. With a higher knowledge level, a producer can master the corresponding operating technology and equipment more easily. As the level of knowledge increases, the cognitive levels of producers will increase and help them to select production technology behaviors that are safer (21, 22). In addition, not only does the accumulation of knowledge help producers to promote technology in the safe production of food and improve their legal awareness of food safety, but it also indirectly loosens the financial constraints and risk constraints faced by producers, creates the necessary conditions for producers to adopt advanced and new technology, and in turn contributes to improving the degree of safety of production technologies. From the perspective of producers, Demazeau et al. (23) examined the factors influencing high-quality production by dairy farmers and found that factors including education level, whether there is technical guidance, and the degree of understanding in food safety knowledge significantly affect safety behaviors of feeding and disinfection. The promotion of production technology and knowledge level is one of the main characteristics in the digital transformation of the industry. A higher knowledge level of the producer leads to greater exertion of the effect of digital technology on the supply side of food. First, it accelerates the circulation and acceptance of safe production technology of food. Second, it strengthens producers' awareness of the safe production of food, prevents food safety hazards by eliminating the root causes, and improves the efficiency of food safety supervision.

H2: A higher knowledge level of food producers strengthens the effect of digital technology on improving the efficiency of food safety supervision.

### Consumer Knowledge Level

Food safety issues ultimately affect the lives and health of consumers. In the process of food safety supervision, consumers play a feedback role, that is, to reflect consumer experience to the market and to regulatory authorities, actively or passively. Barakabitze et al. (24) believe that the higher the consumers' knowledge level, the better their ability to seek out high-quality products in the market, while having a higher awareness of food safety and risk prevention. When consumers encounter food safety issues, they are inclined to disclose such problems publicly or to protect their consumer rights by legal means. Yan et al. (25) also investigated the topic from the consumers' perspective. The study examined the effect of education level on preference for food attributes. The results showed that consumers with higher education and income levels have a stronger ability to obtain information and attach more importance to high-quality labels certified by international agencies. The two studies mentioned show that as the education level increases, consumers' awareness of safe consumption will increase. Moreover, this indicates that when consumers' education level is low, their food safety awareness is also at a low level, and it impedes the effect of digital technology on food safety supervision. Conversely, a higher knowledge level of the consumer leads to a greater effect of digital technology on the consumption side of food. The main manifestation is that a high knowledge level of consumers is conducive to the feedback and supervision effect of digital technology on the end consumer market, thereby enhancing food safety supervision efficiency.

H3: A higher knowledge level of consumers strengthens the effect of digital technology on improving the efficiency of food safety supervision.

## Variable Selection and Data Collection

### Food Supervision Efficiency (Regul_Effic)

Extensive research has been conducted on the evaluation indicator systems of food safety supervision efficiency. For instance, Khayyam et al. (26) believe that the frequency of supervision actions and random inspection can provide a complete reflection of the scale of investments in food safety supervision. The study selected the supervision frequency, random inspection rate of food safety, and rate of administrative punishment as input indicators, and selected the rate of food poisoning and qualified rate of a product as output indicators. Chen et al. (27) selected the average penalty amount and the intensity of the random inspection of food as input indicators and selected the qualified rate of randomly inspected products as an output indicator. The ratio between input and output was used to judge the level of food safety supervision. Kang et al. Kang et al. (28) determined the food safety situation by looking at the number of food poisoning incidents, the number of people affected by food poisonings, the number of deaths from food poisoning, and the qualified rate of random inspected products.

Zhang et al. (29) and Zhang and Song (30) highlighted two shortcomings in the existing methods. First, the effect of food safety supervision funding and other auxiliary supervision equipment on the efficiency of supervision is not duly being considered. Second, the effect of low-quality output on the efficiency of food safety supervision is not duly being considered. These studies have built more comprehensive evaluation indicator systems but have not conducted empirical analyses. Based on the above research, this study constructed an input–output indicator system for food safety supervision efficiency, as shown in Table 1.

**Table 1:** Explanation of the evaluation indicators of food safety supervision efficiency.

	**Indicator**	**Method of calculation**
Input	Level of regulatory funding investment	(Food safety affairs expenditures/total public safety affairs expenditures) × 100%
	Intensity of random inspections	(Total inspected batches/total population of the region) × 100%
	Intensity of administrative punishment	(Total amount of penalty concerning food safety/number of food safety violations) × 100%
Output	Food safety qualified rate	(Qualified batches in random inspections/Total inspected batches) × 100%

Among the above indicators, food safety is a part of public safety affairs, and investment in the supervision of food safety improves supervision efficiency. Sufficient funding of food safety supervision is the key to ensuring the improvement of food safety supervision efficiency; random inspection of products is a direct approach to improve food safety. Generally speaking, the greater the intensity of random inspection per capita, the greater the effect of warnings and food safety supervision. The intensity of random inspection of food is represented by batches inspected per one thousand people in food supervision; the greater the intensity of administrative punishment, the greater the warning and deterrent effect to food producers, which is conducive to promoting the improvement of food production quality. The intensity of administrative punishment is represented by the average amount of the penalty for each food safety violation. The output of food safety supervision refers to the effect of the supervision actions of the food safety supervision departments. The qualified rate of food in random inspection is selected as the output indicator. The higher the qualified rate, the more effective the supervision.

Now that we have explained the input and output indicators, the super-efficiency data envelopment analysis (DEA) method was employed in this study to calculate the efficiency of food safety supervision. This is because first, DEA is a non-parametric method used to evaluate the relative effectiveness of decision-making units (DMUs) under a multi-inputs and multi-outputs mode; and second, when a traditional DEA model is used to calculate the relative efficiency of the DMUs, effective DMUs cannot be further differentiated and compared. To overcome this shortcoming, studies such as Cook et al. (31) and Li et al. (32) proposed and improved the super-efficiency DEA model based on the traditional model so that effective DMUs can also be ranked and compared. The basic idea of the super-efficiency DEA model is that when a DMU is being evaluated, it is excluded from the set of DMUs. As its frontier remains unchanged, the overall efficiency of invalid DMUs is the same as that of the traditional DEA model. For effective DMUs, as its production frontier shifts backwards, the efficiency value obtained will be greater than the measured value in a traditional DEA model, that is, >1. The form of the linear programming is shown in Formula (1) and Formula (2):


(1) $$\begin{array}{l} \min\left[\theta -\tau \left(\overset{m}{\underset{i=1}{\sum }}S_{i}^{-}+\overset{p}{\underset{r=1}{\sum }}S_{r}^{+}\right)\right] \end{array}$$

(2) $$\begin{array}{l} s.t.\{\begin{array}{c} \sum_{j=1,j\neq k}^{n}a_{j}x_{ij}+S_{i}^{-}=\theta x_{ik},\;i=1,2,\ldots ,m\\ \sum_{j=1,j\neq k}^{n}a_{j}x_{rj}-S_{i}^{+}=y_{rk},\;r=1,2,\ldots ,p\\ a_{j}\geq 0,j=1,2,\ldots ,n \end{array} \end{array}$$

θ represents the supervision efficiency under constant returns to scale; $S_{i}^{-}$ and $S_{r}^{+}$ are the slack variables, representing the reduced input and increased output, respectively, *x*_*ij*_ and *y*_*rk*_ represent the input variables and output variables of the model, respectively, *a*_*j*_ is the weight vector of the input factors in the DMU. When θ < 1, it indicates that the supervision of the DMU is not effective; when θ ≥ 1, it indicates that the supervision of the DMU is effective, and the greater the value of θ, the higher the efficiency.

Thirty-one provincial administrative units in China from 2015 to 2017 were taken as the objects of research in this study^2^, while the super-efficiency DEA model was employed to evaluate the supervision efficiency of DMUs. The software EMS 1.3 was used to calculate the super-efficiency value of food safety in each region during 2015, 2016, and 2017.

### Digitalization Level (Digit)

The mean value of indicators included the average rate of fixed broadband ports, mobile phone penetration rate, and mobile Internet penetration rate (33), and the ratio of investment in the telecommunications industry to total investment (34) was selected to represent the level of regional digitalization. These four indicators reflect to a certain extent the regional digital access level, equipment level, application level, and industry development level, respectively. Among these, the average rate of fixed broadband ports, mobile phone penetration rate, and mobile Internet penetration rate are good indicators to represent the level of digital services in a region. To provide a better reflection of the level of digital development in each region, the ratio of industry investment to total industry investment was further adopted as the expression thereof.

### Producer Knowledge Level (Produ_Edu) and Consumer Knowledge Level (Consu_Edu)

Previous studies have shown that the level of education is positively correlated with the individual's cognitive level and knowledge learning ability [e.g., (35–37)]. The higher the level of education, the easier it is to accept the technical products represented by digitization. From the perspective of managers, some studies believe that managers' education level significantly affects the foresight of enterprise strategy (38). Therefore, we can think that the knowledge level of producers and consumers is positively correlated with their education level. In view of this, the knowledge level of food producers in this study is represented by the mean value of the composition ratio of employees in the agriculture, forestry, animal husbandry, and fishery industries with a bachelor's degree or above, and the composition ratio of employees in the food manufacturing industry with a bachelor's degree or above (39). The knowledge level of consumers is expressed by the average education level of the fixed population in each region (40).

### Control Variables

To minimize the potential impact on the results of time-varying regional characteristic variables, control variables such as per capita GDP (Per_GDP), population density (Pop_Dens), and food industry output value (Output_Val) of various countries (41, 42) were added to the calculation to remove the impact of non-critical factors on the efficiency of food safety supervision. In addition, time-fixed effects were used to control the impact of time-varying unobservable factors on the results at the macro level. Individual fixed effects controlled the impact of unobservable factors that are not time varying on the regional-level results.

This study incorporated the direct effects of the level of digital technology on the efficiency of food safety supervision and the moderating effects of knowledge level into the same research framework while considering control variables such as per capita GDP, population density, and food industry output value. On this basis, the direct and indirect effects of digital technology and knowledge level on the efficiency of food safety supervision were examined. The model is shown in Formula (3). Here, α_*i*_ represents the parameters to be estimated, and ε_*i*_, σ_*t*_, and ω_*it*_ are the individual fixed effect, time fixed effect, and random error term, respectively. The meaning of the remaining symbols is the same as above.


(3) $$\begin{array}{l} {Regul\text{\_}Effic}_{it}=\alpha_{0}+\alpha_{1}Digit_{it}+\alpha_{2}{Consu\text{\_}Edu}_{it}\\ \;+\alpha_{3}{Produ\text{\_}Edu}_{it}+\alpha_{4}Digit_{it}{Consu\text{\_}Edu}_{it}\\ \;+\alpha_{5}Digit_{it}{Produ\text{\_}Edu}_{it}+\alpha_{6}{Per\text{\_}GDP}_{it}\\ \;+\alpha_{7}{Pop_{D}ens}_{it}+\alpha_{8}{Output\text{\_}Val}_{it}+\varepsilon_{i}+\sigma_{t}+\omega_{it} \end{array}$$

### Data Source

The data required for this study are all secondary data. (1) The data needed for the calculation of the food supervision efficiency can only be obtained from the official websites of China's provincial Food and Drug Administrations or the Bureau of Statistics website^3^. As the responsible authorities of many regions have not published the latest statistical data or migrated the previous data to new websites, data collection was difficult. Moreover, the statistical calibers and methods of food safety supervision indicators in different regions are inconsistent, leading to problems that included the ineffectiveness of collected data. Therefore, to ensure data accuracy, this study used data from 2015, 2016, and 2017 as the research sample to reduce the impact of such problems as missing samples and inconsistent statistical calibers on the data quality. (2) The sample data of explanatory variables, adjustment variables, and control variables were mainly derived from the China Statistical Yearbook, China Population and Employment Statistical Yearbook, and China's Fixed Asset Investment Statistical Yearbook^4^. The descriptive statistics of the sample data obtained are shown in Table 2.

**Table 2:** Descriptive statistics.

**Variable**	**Obs**.	**Mean**	**St. dev**.	**Min**.	**Max**.
Regul_Effic	93	1.0402	0.5778	0.262	3.426
Digit	93	2.4062	4.5873	0.006	23.5763
Consu_Edu	93	0.089	0.0524	0.04	0.304
Produ_Edu	93	0.0307	0.0294	0.002	0.16
Per_GDP	93	3.3428	1.7624	1.0971	8.4277
Pop_Dens	93	4.2708	6.5533	0.024	37.54
Output_Val	93	7.4339	8.0309	0.0658	44.5199

## Results Analysis and Robustness Test

The test results of the variance inflation factor (VIF) showed no or weak collinearity problems in the model (as shown in Table 3). However, it was expected that adding interaction terms will aggravate the collinearity problems of the model. Regarding existing research methods (43), the core explanatory variables in this study were centralized to reduce the overall collinearity problems of the model, and robust estimation methods were also used to avoid possible heteroscedasticities in the model. On this basis, a fixed-effects model was used to test H1–H3, and the results are shown in Table 4.

**Table 3:** Variance inflation factor test.

**Variables**	**VIF**
Digit	3.18
Consu_Edu	7.45
Produ_Edu	9.60
Per_GDP	6.51
Pop_Dens	1.97
Output_Val	3.48
Mean	5.36

**Table 4:** Digital technology, knowledge level, and food supervision efficiency.

**Variable**	**OLS-Fe**	**POLS**	**OLS-Fe**	**OLS-Re**
Digit_it_	0.09759^***^ (6.88)	0.00018 (0.01)	0.09228^**^ **(2.05)**	0.01157 [0.56]
Consu_Edu_it_	9.04392^*^ (1.97)	2.66346 (0.41)	9.27435^**^ (2.42)	−3.99166 [−0.86]
Produ_Edu_it_	11.8633^**^ (2.24)	0.72685 (0.08)	10.34224^*^ (1.90)	4.06119 [0.74]
Digit_it_×Consu_Edu_it_		0.01219 (0.01)	2.09364^**^ **(2.30)**	0.21780 [0.27]
Digit_it_×Produ_Edu_it_		4.60956^*^ (1.85)	–**1.46757^†^** **(**–**1.56)**	1.95973^†^ [1.53]
Per_GDP_it_	0.19829^**^ (2.22)	0.12972 (1.46)	0.20084^**^ (2.20)	0.30255^***^ [2.96]
Pop_Dens_it_	−0.37289^*^ (−1.85)	−0.04525^**^ (−2.31)	−0.55757^**^ (−2.52)	−0.07381^***^ [−2.91]
Output_Val_it_	−0.01553^*^ (−1.81)	0.01153 (0.55)	−0.00692 (−0.75)	0.00398 [0.33]
Cons	2.08533^**^ (2.23)	0.62881^**^ (2.27)	2.74456^***^ (3.03)	0.26921 [0.93]
Time fixed effect	Yes	No	Yes	No
Regional fixed effect	Yes	No	Yes	No
Hausman test				23.21^***^ <0.0057>
*Q*(*p*)-stat			0.79 <0.374>	
IS-stat			1.63 <0.444>	
*R* ^2^	0.7399	0.1073	0.7577	0.6721
Obs	93	93	93	93

Note: ^†^, ^*^, ^**^, ^***^ indicate that the parameter estimators are significant at the levels of 0.15, 0.10, 0.05, and 0.01, respectively; values in () are the t-values, values in [] are the z-values, and values in < > are the p-values.

*Bold values represent the final reference results*.

The coefficients of determination in Table 4 show that the explanatory power of random effects (OLS-Re) and fixed effects (OLS-Fe) to the model are better than those of the pooled least squares method (POLS). The results of the Hausman test show that the parameter estimators obtained from fixed effects are better than the random effect estimation results, which further proves that the parameter estimators of fixed effects are relatively accurate and reasonable. In addition, the statistics of Q(p)-stat and IS-stat show that the null hypothesis ‘there is no autoregression or serial correlation in the model’ cannot be significantly rejected, which means that there is no spurious regression problem in the model. The above analysis indicates preliminarily that the regression results are reliable and stable to a certain extent.

**Figure 1:**
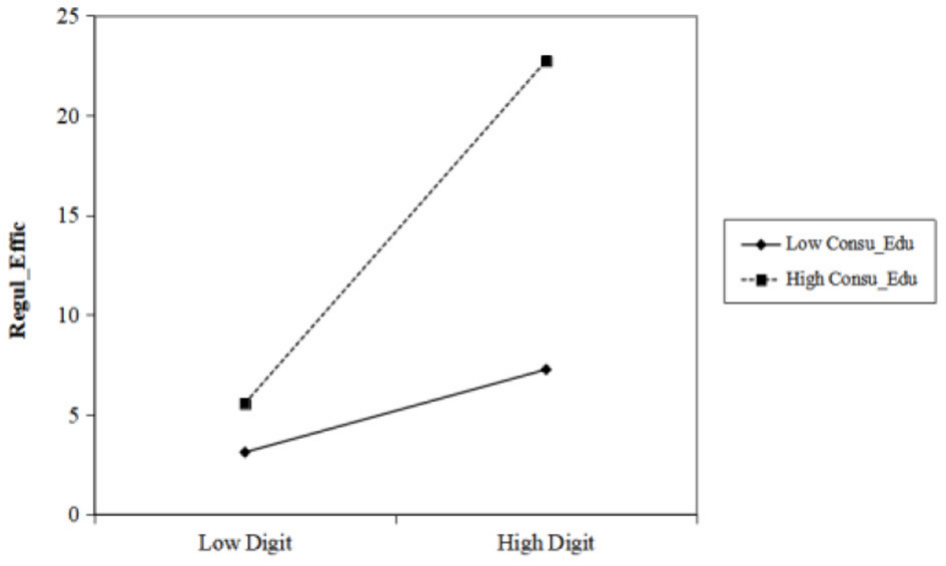
Adjustment effect of consumer knowledge level.

**Figure 2:**
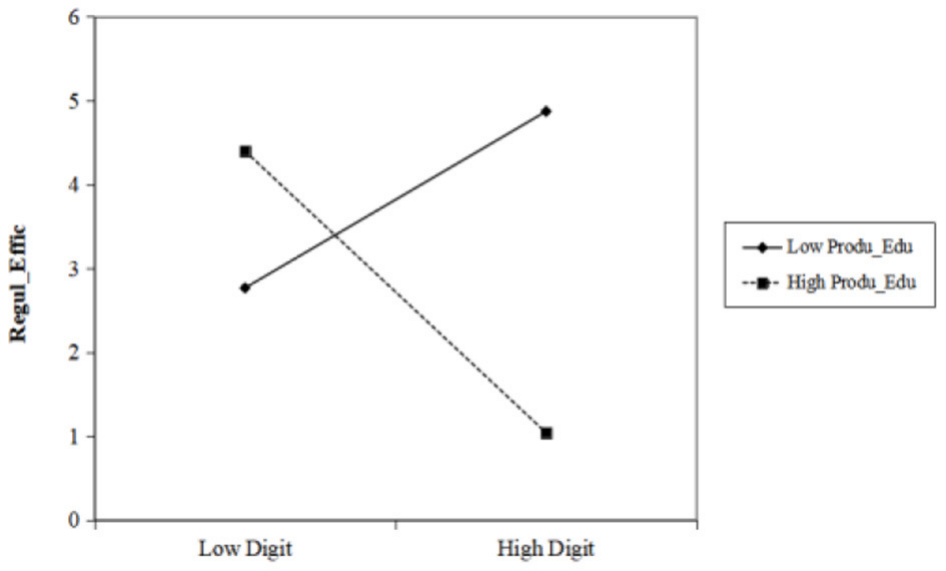
Adjustment effect of producer knowledge level.

The fixed effects regression results show that the parameter estimator of digital technology is 0.09228, and the result is significant at the 5% confidence level, indicating that the level of regional digital technology is positively correlated with the efficiency of food safety supervision, and H1 is confirmed. The parameter estimator of the interaction term of the consumer knowledge level is 2.09364, and the result is significant at the 5% confidence level, indicating that consumer knowledge level has a positive adjustment effect on the improvement of food safety supervision efficiency driven by digital technology and thereby supporting H3. The positive adjustment effect increases as the consumer knowledge level increases, as shown in Figure 1. The parameter estimator of the interaction term of the producer knowledge level is −1.46757. Although the value only reaches the 15% confidence level, its significance is not to be ignored, as the result indicates that producer knowledge level has a negative marginal effect on the improvement of food safety supervision efficiency driven by digital technology, and H2 is not proven (Figure 2). A possible reason is that the education of employees in the food production industry only focuses on technical and business capabilities but has moral and legal education deficiencies. Thus, digital technology might become a tool for some producers who are driven by self-interest to produce food that does not meet the required safety standards, thereby reducing food safety supervision efficiency.

Endogenous problems may occur due to measurement errors or the omission of crucial control variables in the model, leading to inaccurate parameter estimation results. The problems were tested with instrument variables estimation in this study. The similarities and differences between the original parameter estimation results and the parameter estimation results under instrumental variable conditions were compared to determine whether the research conclusion is robust and reliable. The idea of pursuing instrumental variables comes from Porta et al. (44) and Rajan and Zingales (45). When the instrumental variable is difficult to find, the lagged independent variable can also be used as the instrumental variable. Therefore, we attempts to use the digital technology level lagging 1 period as an instrumental variable and uses the two-stage least squares method to test the endogenous problem of the model.

In the process of regression, the dependent variable in the first stage is the digital technology level, while the independent variable is the instrumental variable. Then, the predicted value of the digital technology level in the first stage is brought into the model as an independent variable for regression. The endogenous test results are shown in Table 5. First, the F statistics in the first stage show the correlation between the instrumental variable and the explanatory variable, which indicates a strong instrumental variable. Second, the two columns of parameter estimates represent the results of the second-stage parameter estimation, without the adjustment effect and with the adjustment effect, respectively. The parameter estimator of each model is consistent with the results obtained in Table 4 in terms of the magnitude of the value and significance. Again, the Hausman test results show that, in the case of this model, there is no significant difference between the results of the two-stage least squares method and the fixed-effect estimation method. In other words, it is impossible to reject the null hypothesis ‘there is no estimation bias caused by significant endogenous problems in the model’. Therefore, there are grounds to believe that the model designed in this study and the results obtained are reliable and robust.

**Table 5:** Endogenous test.

**Variable**	**2SLS**	**2SLS**
Digit_it_	0.18117^***^ (11.64)	0.20864^**^ (1.79)
Consu_Edu_it_	13.74281^***^ (3.49)	10.52923^*^ (1.34)
Produ_Edu_it_	4.69192^†^ (2.06)	17.28182^*^ (1.70)
Digit_it_×Consu_Edu_it_		0.62396^†^ (1.31)
Digit_it_×Produ_Edu_it_		−2.00417^†^ (−1.41)
Per_GDP_it_	0.27711^**^ (2.63)	0.25826^***^ (2.29)
Pop_Dens_it_	−0.57782^*^ (−1.68)	−0.55472^*^ (−1.55)
Output_Val_it_	−0.02689^**^ (−2.60)	−0.03529^**^ (−2.25)
Cons	1.03869 (0.74)	0.93208 (0.59)
Time fixed effect	Yes	Yes
Regional fixed effect	Yes	Yes
Hausman test	0.43 <0.9999>	0.61 <0.9999>
*R* ^2^	0.8287	0.8353
Obs	93	93

Note: ^†^, ^*^, ^**^, ^***^ indicate that the parameter estimators are significant at the level of 0.15, 0.10, 0.05, 0.01 respectively; values in () are the t-values, values in < > are the p-values.

To enhance the reliability of our conclusions, a robustness test was conducted after the endogeneity test. Drawing on Heydari et al. (46), we found that digital technology is the basis for promoting informatization development and for improving the application level of informatization. Therefore, Heydari et al. measured the level of regional digitalization from the perspective of informatization investment and used regional informatization density, that is, the ratio between a region's investment in informatization and gross domestic product, as the proxy variable of digitalization. informatization investment usually refers to information and communication technology (ICT) investment, which can be divided into hardware investment and software investment. In this study, fixed investment in electronic information manufacturing was chosen to represent ICT investment in hardware, while fixed investment of the whole society in information transmission, computer services, and the software industry was chosen to represent ICT investment in software. The sum of the two is the total investment in the digital construction of a region.

The first and second columns in Table 6, respectively, represent the parameter estimation results of no interaction item and interaction item added after replacing the sample value. Compared with the results in Table 4, the conclusions presented by the robustness test did not change; hence, the empirical conclusions in this study can be considered robust and reliable.

**Table 6:** Robustness test results.

**Variable**	**OLS-Fe**	**OLS-Fe**
Digit_it_	0.10362^***^ (6.09)	0.09615^**^ (2.20)
Consu_Edu_it_	6.86016^†^ (1.53)	6.15503^*^ (1.67)
Produ_Edu_it_	10.85996^**^ (2.06)	8.58882^*^ (1.72)
Digit_it_×Consu_Edu_it_		2.44427^***^ (2.78)
Digit_it_×Produ_Edu_it_		−1.53999^†^ (−1.54)
Per_GDP_it_	0.10353 (0.64)	0.06206 (0.42)
Pop_Dens_it_	−0.28222 (−1.43)	−0.48052^**^ (−2.23)
Output_Val_it_	−0.02063^*^ (−1.90)	−0.01243 (−1.18)
Cons	1.99785^**^ (2.12)	2.81957^***^ (3.19)
Time fixed effect	Yes	Yes
Regional fixed effect	Yes	Yes
*R* ^2^	0.7553	0.7800
Obs	93	93

Note: ^†^, ^*^, ^**^, ^***^ indicate that the parameter estimators are significant at the level of 0.15, 0.10, 0.05, 0.01 respectively; values in () are the t-values, values in < > are the p-values.

## Discussion

### Summary

Food safety issues have been a focus of attention in various countries. As digital technology gradually penetrates the food industry, what impact does it have on food safety supervision efficiency? How does the adjustment effect of producers' and consumers' knowledge levels influence the relationship between digital technology and food safety supervision efficiency? Answering the two questions above contributes to a fuller understanding of the role of digital technology in food safety supervision and how to maximize the influence of digital technology in improving the efficiency of food safety supervision. Unfortunately, existing literature that focuses on the intrinsic relationship between digital technology and food safety supervision efficiency are limited. To fill the gap left by existing research, this study employed sample data from 31 regions of China between 2015 and 2017 for the empirical analysis of the correlation between the two. At the same time, the moderating effects exerted by the knowledge level of producers and consumers were examined, and the following conclusions were made: The development of digital technology contributes to enhancing the efficiency of food safety supervision. In this process, the higher the knowledge level of consumers, the greater the positive promotional effect of digital technology; on the contrary, when the knowledge level of the producer is higher, it is not conducive to enhancing the effect of digital technology on the efficiency of food safety supervision. The author holds the view that this is related to the fact that employees in the food production and manufacturing industry have insufficient moral and legal knowledge. Not only does this limit the effect of digital technology on enhancing the efficiency of food safety supervision, but this also opens the doors to new ways of illegal production for some unprincipled producers.

### Research Significance

Food safety supervision is not only an important method to protect people's health, but also a prerequisite for maintaining national stability. This is because food is the most basic material condition for people's survival. Food safety issues are related to human life, survival and continuity, and food safety issues usually cause unnecessary burden to a regional medical system. Therefore, in the context of knowledge economy, in order to maintain the public health safety of food, on the one hand, we need to improve the efficiency of food safety supervision and food hygiene quality with the help of digital technology to reduce the occurrence of malignant public health safety events; On the other hand, improve consumers' food safety knowledge and health defense knowledge, improve food producers' food safety production knowledge, and increase the punishment for manufacturers endangering food hygiene and safety.

Overall, this paper studies the relationship between food safety supervision efficiency, digital technology, consumers' and producers' knowledge level of food safety, and tries to interpret the potential relationship between food safety supervision efficiency and public health from the perspective of digitization and knowledge. It is a meaningful research for reducing the occurrence of public health security events and alleviating the pressure of the medical system.

### Policy Implications

In view of the results of this study, the author recommends that food safety supervision work should first apply digital technology to improve supervision efficiency actively. Secondly, increase the moral and legal knowledge of employees in the food production and manufacturing industries through specialized training, regular education, and setting up typical examples. Capitalize on the positive effect of digital technology on food production and processing and reduce the negative effects. Finally, encourage consumers to make use of digital technology to assist the regulatory authorities in supervising food safety issues and to regulate the production behavior of producers in as many ways as possible.

### Limitation and Future Research

Due to restricted data availability, this study only employed data from China as an example in the empirical analysis and did not include data from other countries or regions. This limitation will be addressed in follow-up research.

## Data Availability Statement

The original contributions presented in the study are included in the article/supplementary material, further inquiries can be directed to the corresponding author/s.

## Author Contributions

All authors undertook research, writing, and review tasks throughout this study. All authors have read and agreed to the published version of the manuscript.

## Funding

This research was funded in part by the Major Program of the National Social Science Foundation of China [grant number 20&ZD124], the National Social Science Foundation of China [grant number 21CJY024], the National Natural Science Foundation of China [grant numbers 71773115, 72174180, 72074195, 71973129, 72072162, and 72173073], the Philosophy and Social Science Program of Zhejiang [grant numbers 22NDQN290YB, 22QNYC13ZD, and 21NDYD097Z], the Humanity and Social Science Foundation of Ministry of Education of China [grant numbers 21YJA790043, 21YJA630037, 19YJA790107, 19YJA630092, 18YJA790088, and 21YJCZH213] and the Natural Science Foundation of Shandong [grant number ZR2020MG060].

## Conflict of Interest

The authors declare that the research was conducted in the absence of any commercial or financial relationships that could be construed as a potential conflict of interest.

## Publisher's Note

All claims expressed in this article are solely those of the authors and do not necessarily represent those of their affiliated organizations, or those of the publisher, the editors and the reviewers. Any product that may be evaluated in this article, or claim that may be made by its manufacturer, is not guaranteed or endorsed by the publisher.

## Footnotes

^1^Information extracted from the food safety data published by the World Health Organization: https://www.who.int/news-room/fact-sheets/detail/food-safety.

^2^Considering the completeness and accessibility of the data, in this study, the data of 31 regions from 2015 to 2017 were used. As the data in Hong Kong, Macau, and Taiwan were insufficient, these regions were not included in the research.

^3^For example, the data in Beijing comes from the information published on the website of the Beijing Municipal Medical Products Administration (http://yjj.beijing.gov.cn/yjj/xxcx/zlgg/yp30/index.html).

^4^All yearbook data comes from China's economic and social big data platform (https://data.cnki.net/?al=f).

## References

[1] Scharff RLS. State estimates for the annual cost of food borne illness. J Food Protect. (2015) 78:1064. 10.4315/0362-028X.JFP-14-505 26038894

[2] Yuan JJ Lu YL Cao XH Cui HTR. wildlife conservation and food safety to prevent human exposure to novel virus. Ecosyst Health Sust. (2020) 6:1741325. 10.1080/20964129.2020.1741325

[3] Chanchitpricha C Bond A. Conceptualising the effectiveness of impact assessment processes. Environ Impact Assess Rev. (2013) 43:65–72. 10.1016/j.eiar.2013.05.006

[4] Herman PM Mahrer NE Wolchik SA Porter MM Jones S Sandler IN. Cost-benefit analysis of a preventive intervention for divorced families: reduction in mental health and justice system service use costs 15 years later. Prev Sci. (2015) 16:586–96. 10.1007/s11121-014-0527-6 25382415 PMC6112169

[5] Traill WB Koenig A. Economic assessment of food safety standards: costs and benefits of alternative approaches. Food Control. (2010) 21:1611–9. 10.1016/j.foodcont.2009.06.018

[6] Millstone E Lang T Marsden T. Food brexit and chlorinated chicken: a microcosm of wider food problems. Polit Quart. (2019) 90:645–53. 10.1111/1467-923X.12780

[7] Han GH Yan S. Does food safety risk perception affect the public's trust in their government? An empirical study on a national survey in China. Int J Environ Res Public Health. (2019) 16:1874. 10.3390/ijerph16111874 31141881 PMC6603658

[8] Smythies J Lantremange MDDT. nature and function of digital information compression mechanisms in the brain and in digital television technology. Front Syst Neurosci. (2016) 10:40. 10.3389/fnsys.2016.00040 27199688 PMC4858531

[9] Di Giacomo D. Vittorini D Lacasa P Editorial P. Digital skills and Life-long learning: digital learning as a new insight of enhanced learning by the innovative approach joining technology and cognition. Front. Psychol. (2018) 9:2621. 10.3389/fpsyg.2018.02621 30631297 PMC6315150

[10] Nakasone E Torero M. A text message away: ICTs as a tool to improve food security. Agric Econ. (2016) 47:49–59. 10.1111/agec.12314

[11] Omulo G Kumeh EM. Farmer-to-farmer digital network as a strategy to strengthen agricultural performance in Kenya: a research note on 'Wefarm' platform. Technol Forecast Soc Change. (2020) 158:120–120. 10.1016/j.techfore.2020.120120

[12] Cranfield J Blandon J Henson S. Small-scale farmer participation in new agri-food supply chains: case of the supermarket supply chain for fruit and vegetables in Honduras. J Int Dev. (2010) 21:971–84. 10.1002/jid.149025855820

[13] Awuor F Raburu G Onditi A Rambim D. Building e-agriculture framework in Kenya. J Agric Informat. (2016) 7:75–93. 10.17700/jai.2016.7.1.244

[14] Shinwell J Defeyter MA. Food insecurity: a constant factor in the lives of low-income families in Scotland and England. Front Public Health. (2021) 9:588254. 10.3389/fpubh.2021.588254 34095040 PMC8170021

[15] Reeder N Tapanee P Persell A Tolar-Peterson T. Food insecurity, depression, and race: correlations observed among college students at a university in the Southeastern United States. Int J Environ Res Public Health. (2020) 17:8268. 10.3390/ijerph17218268 33182386 PMC7664923

[16] Marcotrigiano V Cinquetti S Flamini R De Rosso M Ferraro L Petrilli S. Safety in wine production: a pilot study on the quality evaluation of prosecco wine in the framework of UE regulation. Int J Environ Res Public Health. (2020) 17:3283. 10.3390/ijerph17093283 32397210 PMC7246455

[17] Cairns G. Evolutions in food marketing, quantifying the impact, and policy implications. Appetite. (2013) 62:194–7. 10.1016/j.appet.2012.07.016 22858428

[18] Dubé L Labban A Moubarac JC Heslop G Ma Y Paquet C. A nutrition/health mindset on commercial Big Data and drivers of food demand in modern and traditional systems. Ann New York Acad Sci. (2015) 1331:278–95. 10.1111/nyas.12595 25514866

[19] Qiu F Hu Q Xu B. Fresh agricultural products supply chain coordination and volume loss reduction based on strategic consumer. Int J Environ Res Public Health. (2020) 17:7915. 10.3390/ijerph17217915 33126663 PMC7663347

[20] Dubé L Mcrae C Wu YH Ghosh S Allen S Ross D. (2020). Impact of the eKutir ICT-enabled social enterprise and its distributed micro-entrepreneur strategy on fruit and vegetable consumption: a quasi-experimental study in rural and urban communities in Odisha, India. Food Policy. (2020) 90:101787. 10.1016/j.foodpol.2019.101787

[21] Hu F Xi X Zhang Y. Influencing mechanism of reverse knowledge spillover on investment enterprises' technological progress: an empirical examination of Chinese firms. Technol Forecast Soc. (2021) 169:120797. 10.1016/j.techfore.2021.120797

[22] Irene B-G Varela-Ortega C Manners R. Evaluating animal-based foods and Plant-based alternatives using multi-criteria and SWOT analyses. Int J Environ Res Public Health. (2020) 17:7969. 10.3390/ijerph17217969 33138318 PMC7662315

[23] Demazeau G Plumecocq A Lehours P Martin P Couëdelo L Billeaud C. A new high hydrostatic pressure process to assure the microbial safety of human milk while preserving the biological activity of its main components. Front Public Health. (2018) 6:306. 10.3389/fpubh.2018.00306 30460221 PMC6232532

[24] Barakabitze AA Kitindi EJ Sanga C Shabani A Philipo J Kibirige G. New technologies for disseminating and communicating agriculture knowledge and information: challenges for agricultural research institutes in Tanzania. Electron J Inf Syst Dev Ctries. (2015) 70:1–22. 10.1002/j.1681-4835.2015.tb00502.x25855820

[25] Yan B Fan J Cai C. Fang J. Supply chain coordination of fresh Agri-products based on value loss. Oper Manag Res. (2020) 13:185–96. 10.1007/s12063-020-00162-z

[26] Khayyam M Chuanmin S Qasim H Ihtisham M Anjum R Jiaxin L. et al. Food consumption behavior of Pakistani students living in China: the role of food safety and health consciousness in the wake of coronavirus disease 2019 Pandemic. Front Psychol. (2021) 12:673771. 10.3389/fpsyg.2021.673771 34385954 PMC8353093

[27] Chen T Wang L Wang J. Transparent assessment of the supervision information in China's food safety: a fuzzy-ANP comprehensive evaluation method. J Food Quality. (2017) 2017:1–14. 10.1155/2017/4340869

[28] Kang S Ho TTT Lee N-J. Comparative studies on patient safety culture to strengthen health systems among southeast Asian countries. Front Public Health. (2021) 8:600216. 10.3389/fpubh.2020.600216 33511097 PMC7835724

[29] Zhang X Zhang J Chen T. An ANP-fuzzy evaluation model of food quality safety supervision based on China's data. Food Sci Nutr. (2020) 8:3157–63. 10.1002/fsn3.1561 32724580 PMC7382133

[30] Zhang Y Song YH. Identification of food safety risk factors based on intelligence flow and DEMATEL-ISM. DYNA. (2020) 95:418–24. 10.6036/9636

[31] Cook WD Liang L Zha Y Zhu J. A modified super-efficiency DEA model for infeasibility. J Oper Res Soc. (2009) 60:276–81. 10.1057/palgrave.jors.2602544

[32] Li Z Tian Y Gong Z Qian L. Health literacy and regional heterogeneities in China: a population-based study. Front Public Health. (2021) 9:603325. 10.3389/fpubh.2021.603325 34046382 PMC8144299

[33] Scheerder A Deursen AV Dijk JV. Determinants of internet skills, uses and outcomes. A systematic review of the second- and third-level digital divide. Telemat Inform. (2017) 34:1607–24. 10.1016/j.tele.2017.07.007

[34] Szeles MR. New insights from a multilevel approach to the regional digital divide in the European Union. Telecommun Policy. (2018) 42:452–63. 10.1016/j.telpol.2018.03.007

[35] Jung T Ejermo O. Demographic patterns and trends in patenting: gender, age, and education of inventors. Technol Forecast Soc. (2014) 86:110–24. 10.1016/j.techfore.2013.08.023

[36] Tegegne GT Kefale B Engidaw MT Degu A Tesfa D Ewunetei A. Knowledge, attitude, and practice of healthcare providers toward novel coronavirus 19 during the first months of the pandemic: a systematic review. Front Public Health. (2021) 9:606666. 10.3389/fpubh.2021.606666 34249826 PMC8267791

[37] De-la-Pena C Luque-Rojas MJ. Levels of reading comprehension in higher education: systematic review and meta-analysis. Front. Psychol. (2021) 12:712901. 10.3389/fpsyg.2021.712901 34421765 PMC8371198

[38] Hazelzet E Bosma H de-Rijk A Houkes I. Does dialogue improve the sustainable employability of low-educated employees? A study protocol for an effect and process evaluation of “Healthy HR”. Front Public Health. (2020) 8:446. 10.3389/fpubh.2020.00446 33014964 PMC7505925

[39] Isin S Yildirim I. Fruit-growers' perceptions on the harmful effects of pesticides and their reflection on practices: the case of Kemalpasa, Turkey. Crop Prot. (2007) 26:917–22. 10.1016/j.cropro.2006.08.006

[40] Parente ST Salkever DS Davanzo J. The role of consumer knowledge on the demand for preventive health care among the elderly. Health Econ. (2005) 14:25–38. 10.1002/hec.907 15386668

[41] Piggott NE Marsh TL. Does food safety information impact U.S. meat demand? Am. J Agr Econ. (2004) 86:154–74. 10.1111/j.0092-5853.2004.00569.x25927118

[42] Wang J Diao H Tou L. Research on the influence mechanism of rational consumers' food safety supervision satisfaction. Int J Environ Res Public Health. (2019) 16:739. 10.3390/ijerph16050739 30823657 PMC6427581

[43] Lennox CS Francis JR Wang Z. Selection models in accounting research. Account Rev. (2012) 87:589–616. 10.2308/accr-10195

[44] Porta RL Lopez-De-Silanes F Shleifer A Vishny RW. Legal determinants of external finance. J Financ. (1997) 52:1131–50. 10.1111/j.1540-6261.1997.tb02727.x12294993

[45] Rajan RG Zingales L. Financial dependence and growth. Am Econ Rev. (1998) 88:559–86.

[46] Heydari M Lai KK Zhou X. How to manage red alert in emergency and disaster unit in the hospital? Evidence from London. Front. Public Health. (2021) 9:634417. 10.3389/fpubh.2021.634417 34621713 PMC8490805

